# Acute suppurative thyroiditis with transient hyperthyroidism in thalassemia child: an uncommon association in a common disease—a rare case report

**DOI:** 10.1186/s43054-020-00035-x

**Published:** 2020-07-13

**Authors:** Swaranjika Sahoo, Basudev Biswal, Anuspandana Mohapatra, Rachita Sarangi

**Affiliations:** Department of Pediatrics, IMS and SUM Hospital, SOA Deemed to be University, Bhubaneswar, Orissa 751003 India

**Keywords:** Acute suppurative thyroiditis, Hyperthyroidism, Thalassemia, Case report

## Abstract

**Background:**

Thyroid infection is rare in paediatric age group in anatomically normal thyroid gland. However, acute suppurative thyroiditis (AST) is common in high-risk cases like thyroglossal duct remnant and thyroglossal pyriform sinus fistula. This may be associated with variable thyroid dysfunctions. Though transient hyperthyroidism is reported in some cases, its association was not documented in thalassemia child.

**Case presentation:**

We are reporting an 8-year-old male child with a history of fever, pain, and left neck swelling for 5 days. He was evaluated and empirically started intravenous antibiotics (ceftriaxone, cloxacillin, and amikacin) along with other supportive treatment for localized signs of thyroid infection. Ultrasonography of the neck revealed intercommunicating pockets of collections with internal echoes in the left thyroid lobe. CT neck was done and suggested that the left lobe of the thyroid gland was replaced by hypodense lesions of fluid attenuation without any congenital abnormality of the thyroid gland. His thyroid function tests were suggestive of biochemical hyperthyroidism. He was treated with intravenous antibiotics and ultrasonography-guided fine-needle aspiration of pus. His pus culture showed growth of methicillin resistant *Staphylococcus aureus*. He also had microcytic hypochromic anaemia which was evaluated and found to have thalassemia trait. His biochemical hyperthyroidism normalized after completion of 2 weeks of treatment and required no intervention.

**Conclusion:**

Acute suppurative thyroiditis is an uncommon clinical condition in an anatomically normal thyroid gland which could be associated with thyroid dysfunction. Its association in a thalassemia child is also not documented in literature. Clinical evaluation and proper history taking and non-invasive thyroid imaging are the cornerstone for diagnosis. Antibiotics and pus drainage are the mainstay management. Associated biochemical hyperthyroidism is transient and resolves without any specific medication in asymptomatic cases.

## Background

Thyroid gland is an organ with a remarkable resistance to infection due to its profound vascular and lymphatic supply [1]. Fibrous encapsulation of the thyroid gland, separating it from other structures of the neck with abundance of iodine and hydrogen peroxide prevents growth of microorganisms [1]. Acute suppurative thyroiditis (AST) is thus an uncommon presentation in the paediatric age group [2]. Less than 0.7% of cases present as AST among thyroid disorders which may present with fever and painful neck swelling [3]. AST can be associated with thyroid dysfunctions which normalizes after management of infection [4].

## Case presentation

**Fig. 1:**
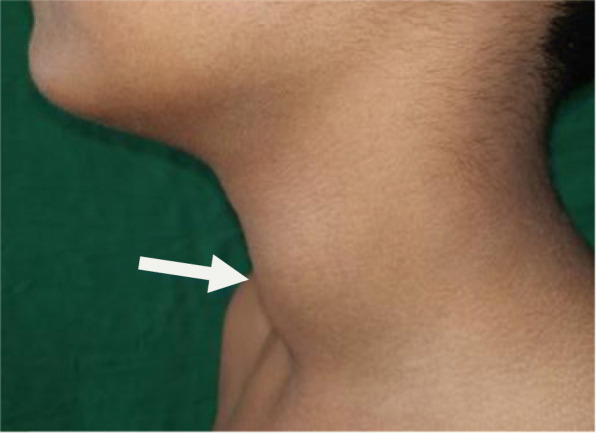
Picture depicting swelling of neck over left side (white arrow)

An 8-year-old boy presented with high-grade fever, pain, and swelling of left side of neck region since 5 days. Child has positive consanguinity with no family history of any haemolytic anaemia. His body weight was 25 kg (25th–50th centile) and height was 125 cm (25th–50th centile) as per Indian Academy of Pediatrics growth chart. His temperature was 101.8° F, heart rate 102/min, respiratory rate 24/min, and blood pressure 100/76 mmHg. On local examination, he had a soft, tender, fluctuant swelling of size 2 cm × 2 cm noted in the left lobe of the thyroid gland (Fig. 1) with no local pus discharge and with regional cervical lymphadenopathy. Other systemic examinations were normal.

**Fig. 2:**
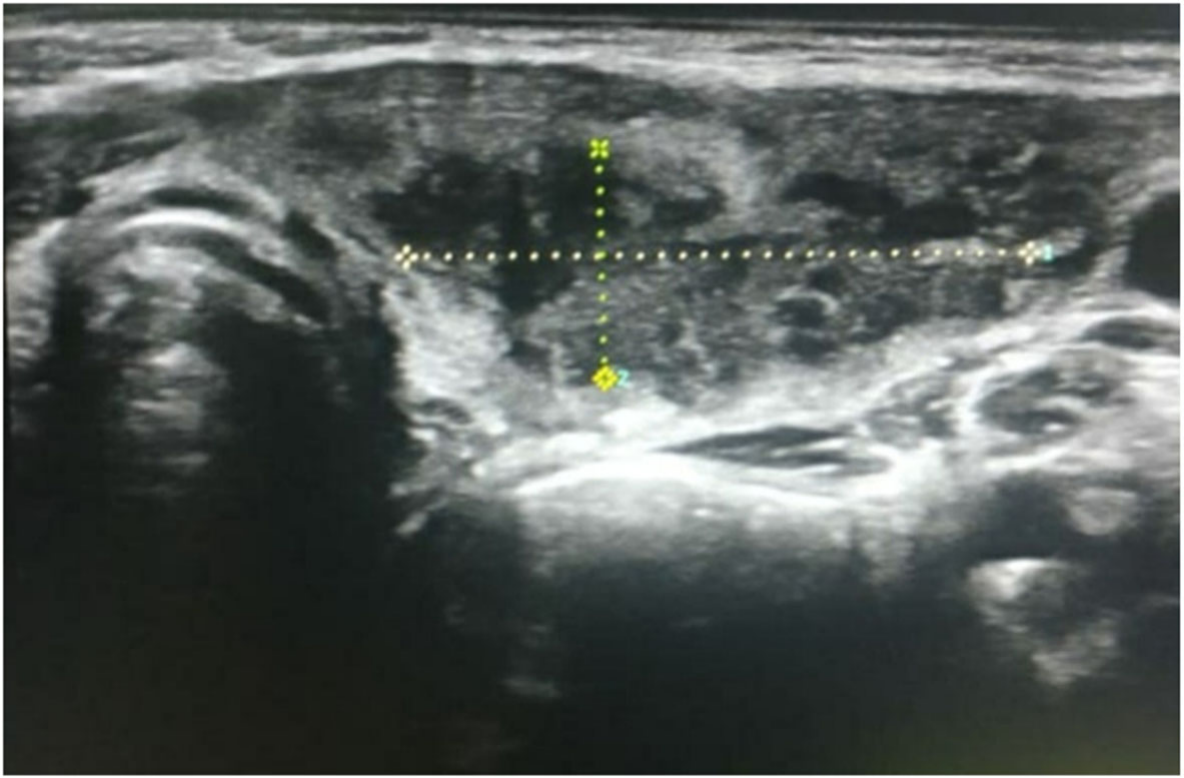
Ultrasonography of neck suggestive of intercommunicating pockets of collections with internal echoes in left thyroid lobe (demarcated dotted area)

**Fig. 3:**
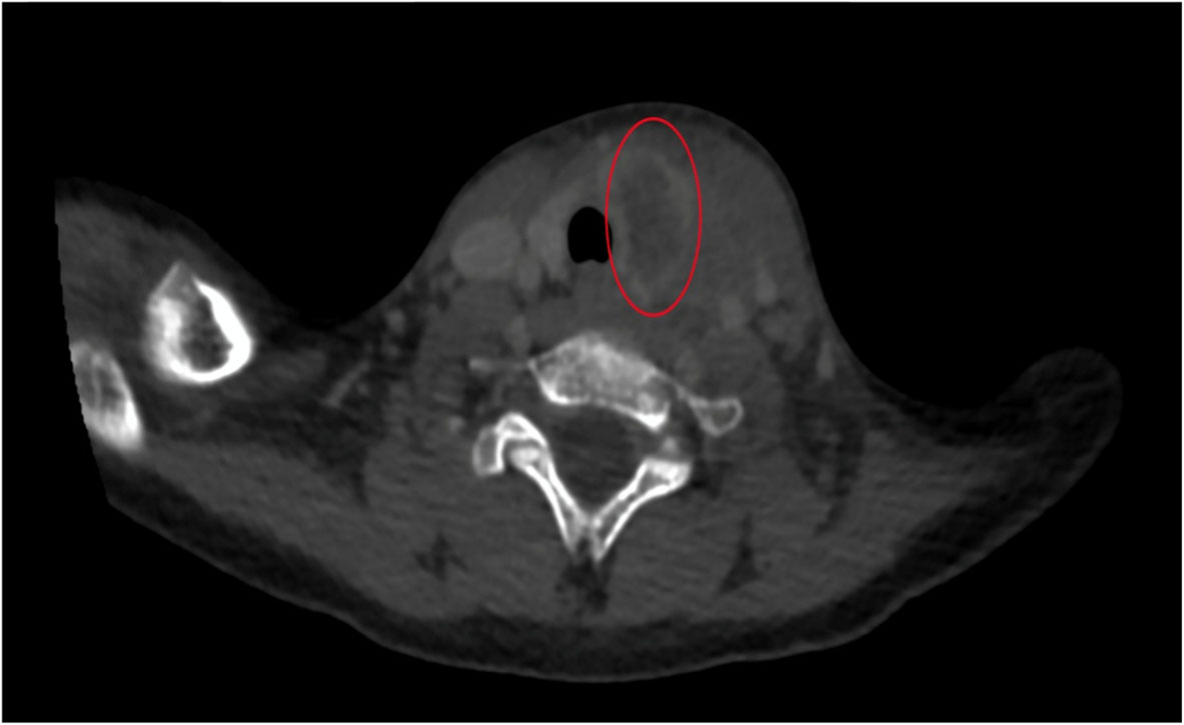
CT scan of neck depicting left lobe of thyroid replaced by hypodense lesions of fluid attenuation (red demarcated circle area); features suggestive of acute suppurative thyroiditis

Initial laboratory investigations showed a haemoglobin level of 102 g/L, mean corpuscular volume of 64 fl, total leucocyte count of 15.84 × 10^9^/L with neutrophilia (82.6%), Peripheral blood smear was suggestive of microcytic hypochromic anaemia with anisocytosis, poikilocytosis with neutrophilic leucocytosis. Total platelet count was 275 × 10^9^/L and C-reactive protein 33.5 μg/L. Ultrasonography of neck was suggestive of intercommunicating pockets of collections with internal echoes in left thyroid lobe with reactive left cervical lymph nodes (Fig. 2).Thyroid function tests were suggestive of hyperthyroidism [triiodothyronine 2.26 nmol/L (2.01–3.12 nmol/L), thyroxine 193.98 nmol/L (73.79–151.06 nmol/L), and thyroid-stimulating hormone 0.02 mIU/L (0.55–5.31 mIU/L)]. The anti-thyroid peroxidase (TPO) antibody test was negative. His haemoglobin electrophoresis reports were suggestive of beta thalassemia trait (HbA 95.4%, HbA_2_ 4.6%). Parents were evaluated for thalassaemia and only the mother was found to be beta thalassaemia trait. Child was started empirically on intravenous antibiotics (ceftriaxone, cloxacillin, and amikacin) along with other supportive treatment (paracetamol and serratiopeptidase). Contrast-enhanced computed tomography (CECT) of neck was done and was suggestive of thyroid abscess localized to left lobe without any congenital abnormality (Fig. 3). Ultrasonography-guided fine-needle aspiration of thyroid swelling was done and 3 cc of pus was collected and sent for Gram stain, acid fast bacilli stain, pus culture, and sensitivity. Pus culture yielded growth of methicillin resistant *Staphylococcus aureus*. In view of culture sensitivity report, injection cloxacillin was switched to injection of linezolid. Repeat thyroid function tests were normal after 2 weeks of antibiotic therapy and no intervention was started for transient hyperthyroidism.

## Discussion

AST is associated with congenital anomalies of the 3rd and 4th brachial arch in 2–8% of cases [2]. Patient usually presents with fever, painful swelling of neck, and rarely dysphasia, dysphonia [5]. AST can be associated with euthyroidism, hypothyroidism, and rarely hyperthyroidism [4, 6]. Hypothyroidism is also well documented in children with beta thalassaemia major [7]. However, we could not document association of transient hyperthyroidism in a thalassaemia child with AST after a profound literature search. Ultrasonographic study guides for the diagnosis and CT scan delineates underlying malformation, status of spread, and fistula formation [2]. The common infections causing AST are *Staphylococcus aureus* and *Streptococcus pneumoniae*, rarely *Salmonella*, *Escherichia coli*, and *Haemophilus influenzae* [3, 8, 9]. Primary modes of treatment are intravenous antibiotics and individualized surgical interventions like fine-needle aspiration or incision and pus drainage [9]. Most of the patients become asymptomatic with intravenous antibiotics, while some of them need ultrasonography-guided fine-needle aspiration [9]. Thyroid dysfunction during AST is transient and repeated testing of thyroid function should be done during the course of treatment [4]. As biochemical thyroid dysfunction during AST may resolve, active treatment should be reserved for symptomatic cases [4]. Though this is a single case report, a prospective study of similar cases would guide regarding their clinical presentation and management.

## Conclusion

AST is an uncommon clinical condition in anatomically normal thyroid gland, which could be associated with thyroid dysfunction. AST in a child without any congenital malformation is also rarely documented [2] and its association with thalassemia is not documented .Clinical history and non-invasive thyroid imagings are the cornerstones for diagnosis [10]. Antibiotics and pus drainage are the mainstays of management [9, 10]. AST-associated biochemical hyperthyroidism is transient and resolves after treatment [4].

## Data Availability

Not applicable

## Abbreviations

AST: Acute suppurative thyroiditis
TPO: Thyroid peroxidase
CECT: Contrast-enhanced computed tomography
